# Neurophysiological Indicators of Residual Cognitive Capacity in the Minimally Conscious State

**DOI:** 10.1155/2015/145913

**Published:** 2015-10-04

**Authors:** Solveig L. Hauger, Caroline Schnakers, Stein Andersson, Frank Becker, Torgeir Moberget, Joseph T. Giacino, Anne-Kristine Schanke, Marianne Løvstad

**Affiliations:** ^1^Department of Research, Sunnaas Rehabilitation Hospital, 1450 Nesoddtangen, Norway; ^2^Department of Psychology and Neurosurgery, University of California, Los Angeles, CA 90095-1563, USA; ^3^Department of Psychology, University of Oslo, Postboks 1094 Blindern, 0317 Oslo, Norway; ^4^Institute of Clinical Medicine, University of Oslo, Postboks 1171 Blindern, 0318 Oslo, Norway; ^5^Norwegian Centre for Mental Disorders Research (NORMENT), KG Jebsen Centre for Psychosis Research, Division of Mental Health and Addiction, Oslo University Hospital, Postboks 4953 Nydalen, 0424 Oslo, Norway; ^6^Regional Center of Knowledge Translation in Rehabilitation, Sunnaas Rehabilitation Hospital, 1450 Nesoddtangen, Norway; ^7^Department of Physical Medicine and Rehabilitation, Harvard Medical School and Spaulding Rehabilitation Hospital, 300 First Avenue, Charlestown, MA 02129, USA

## Abstract

*Background*. The diagnostic usefulness of electrophysiological methods in assessing disorders of consciousness (DoC) remains to be established on an individual patient level, and there is need to determine what constitutes robust experimental paradigm to elicit electrophysiological indices of covert cognitive capacity. *Objectives*. Two tasks encompassing active and passive conditions were explored in an event-related potentials (ERP) study. The task robustness was studied in healthy controls, and their utility to detect covert signs of command-following on an individual patient level was investigated in patients in a minimally conscious state (MCS). *Methods*. Twenty healthy controls and 20 MCS patients participated. The active tasks included (1) listening for a change of pitch in the subject's own name (SON) and (2) counting SON, both contrasted to passive conditions. Midline ERPs are reported. *Results*. A larger P3 response was detected in the counting task compared to active listening to pitch change in the healthy controls. On an individual level, the counting task revealed a higher rate of responders among both healthy subjects and MCS patients. *Conclusion*. ERP paradigms involving actively counting SON represent a robust paradigm in probing for volitional cognition in minimally conscious patients and add important diagnostic information in some patients.

## 1. Introduction

A minority of patients with severe acquired brain injury remain in a state of disordered consciousness (DoC) after awakening from coma [1, 2], and some experience prolonged DoC [3, 4]. Following coma, most patients transition to either a vegetative state (VS), also referred to as the “unresponsive wakefulness syndrome (UWS)” [5], or a minimally conscious state (MCS). While VS is characterized by intermittent wakefulness in the absence of any behavioral signs of awareness, MCS is characterized by the presence of inconsistent, but clearly discernible behavioral evidence of awareness of self or the environment (i.e., visual pursuit, localization to pain, or reproducible command-following) [6]. It has recently been suggested that MCS can be subcategorized into MCS+ and MCS−, based on the presence or absence of language function. MCS+ is defined by the presence of command-following, intelligible verbalization, or gestural or verbal yes/no responses. In contrast, MCS− is characterized by nonlinguistic signs of conscious awareness such as visual pursuit and localization of noxious stimuli or other behaviors that selectively occur in response to specific stimuli (e.g., appropriate smiling or crying to a picture of a family member) [7].

Adequate assessment of level of consciousness is challenging, but crucial, in establishment of an appropriate plan of care and provision of an accurate prognosis to caregivers and may affect end-of-life decisions [8]. Importantly, patient, examiner, and environmental factors have been recognized as sources of inaccurate diagnosis in DoC patients [9], and rates of misdiagnosis have been reported to be up to ~40% [10, 11]. Even experienced clinicians can be mistaken when differentiation between volitional and reflexive behavior is based on unstructured bedside examinations instead of standardized assessment procedures [12–14]. Standardized behavioral assessment is the most common diagnostic method, and the Coma Recovery Scale-Revised (CRS-R) has been recommended as the preferred assessment scale [13]. Standardized measures depend on the patient's ability to move and communicate; however, conscious awareness may be masked as the result of severe sensory and motor deficits [15]. The diagnostic validity of behavioral assessment may be compromised by these issues.

Over the last two decades, neurodiagnostic techniques have been explored that do not rely on overt behavioral responses. Included among these are techniques designed to detect patterns of brain activity such as event-related brain potentials (ERPs) and functional magnetic resonance imaging (fMRI). Functional imaging and neurophysiological studies have shown that when using standardized diagnostic scales, like the CRS-R, volitional behaviors such as command-following may go undetected in a minority of patients [16–19], suggesting a key role for fMRI and electrophysiological studies in detecting covert cognition in patients with DoC.

ERP is a promising, low-cost, noninvasive technique that can be conducted repeatedly at bedside [20, 21]. Event-related potentials (ERPs) are extracted from continuous electroencephalography (EEG) that is recorded from the skull while participants are exposed to repeated stimulus presentations in cognitive tasks. Signal averaging is used to eliminate the background EEG activity to derive an averaged measure of stimulus-related processing [21]. Thus, ERPs represent time-locked EEG activity elicited by external events, providing a neurophysiological correlate of cognitive processing at the millisecond level, from early and largely sensory components, that is, the N1 component, to later and cognitively mediated waveforms, such as the P3. The well-established P3 component has attracted particular interest in the DoC population, as it reflects allocation of attentional resources [22, 23], and presence of the P3 wave is a reliable predictor of awakening from coma [24]. In a clinical context, it is essential to understand to what degree ERPs may add valuable clinical information on an individual level and, furthermore, under what experimental conditions this can be best done. It has been argued that ERP experiments need to include subjectively meaningful stimuli, as the probability of electrophysiological responses in DoC patient increases with salient self-referential stimuli [25]. The salient value of the person's own name (SON) has proven promising in eliciting the P3 response [26–29], even when repeated extensively [30]. SON also seems to result in enhanced responses in healthy and awake subjects [31–33] and during sleep [33, 34], implying that SON is a robust salient stimulus. However, the inference of consciousness based on passive ERP paradigms is insufficient, as passive tasks without demand of volitional mental effort can elicit a P3 response in comatose or VS patients [26, 28] and in healthy subjects under anaesthesia [35]. Therefore, a second argument concerns the necessity to include “active” experimental paradigms requiring volitional cognitive effort, allowing detection of covert command-following by comparing P3 response in passive versus active tasks [30, 36–38].

In their ERP study, Schnakers et al. [38] presented a list of eight randomized names, including SON. When instructed to actively count a target name (either SON or an unfamiliar name (UN)), the MCS, but not VS group, showed an increase in P3 amplitude. The study reported that 9/14 individual MCS patients had enhanced P3 amplitudes in one out of two active counting conditions, but 2/8 patients showing command-following at bedside did not show the suspected ERP (false negatives). Also, covert command-following was detected in two MCS patients with absence of externally observable signs of command-following. In a more recent study, the Schnakers et al. [38] paradigm was developed into a single-stimuli paradigm, presenting SON in a passive listening condition along with an active condition, instructing patients to listen for a change of pitch in the voice saying their name. They found that 5/8 MCS+ patients and 3/8 MCS− patients versus only 1/10 VS patients displayed enhanced P3 amplitude in the active versus passive condition [30]. Other studies using active ERP paradigms have also demonstrated signs of covert volitional mental effort in DoC [36, 37]. Despite increased knowledge of ERP responses typical for DoC patients at a group level, the literature is still sparse, and little is known about what type of cognitive task constitutes the most robust paradigm in order to elicit electrophysiological indices of covert cognitive capacity. Some studies report only group level findings or lack sufficient reports of false negative rates [36, 37, 39]. Thus, the potential clinical utility of electrophysiological methods on an individual patient level remains largely unknown. An important step forward is therefore establishing paradigms that are recognized as robust in healthy individuals. Further, the efficacy of these paradigms in revealing covert voluntary cognition on an individual patient level needs to be explored. This is paramount in order to establish the diagnostic utility of the ERP method in clinical practice, where correct assessment of a DoC patient's level of consciousness is crucial. Thus, reproducing the results from active paradigms is necessary in order to recognize their value and limitations.

The present study aims to replicate and expand upon previous ERP study designs [30, 38]. We specifically wished to explore the robustness of a paradigm involving both passive and active conditions using SON. The aim of the study was twofold. The first aim was to investigate the robustness of two separate active tasks with varying stimulus type and cognitive load in healthy controls. It was expected that the salient value of SON would elicit more pronounced responses compared to an unfamiliar name (UN). It was furthermore anticipated that SON would elicit a larger P3 in active compared to passive tasks. It was also an aim to explore the rate of healthy controls with enhanced P3 in the two active compared to passive conditions. The second aim was to compare the MCS+ and MCS− patients with regard to the amplitude of the P3 in the active versus passive conditions. It was expected that more patients in the MCS+ group would demonstrate an enhanced P3 in the active conditions than in the MCS− group. We also anticipated that electrophysiological indications of command-following would be observed in a minority of MCS− patients and that some MCS+ patients would fail to display P3 in the active conditions.

## 2. Materials and Methods

### 2.1. Participants

Twenty-two healthy controls aged 18–65 years were enrolled in the study. All were native Norwegian speakers with no previous history of brain injury, neurological or psychiatric illness, premorbid hearing impairments, or cognitive deficits. Health personnel at Sunnaas Rehabilitation Hospital were recruited as healthy controls. Twenty-two patients were enrolled from the Brain Injury Unit at Sunnaas Rehabilitation Hospital in Oslo, Norway, and two patients from St. Olavs Hospital in Trondheim. All were above 18 years of age and were fluent Norwegian speakers prior to their injury. Patients were assessed with the CRS-R and met the diagnostic criteria for MCS [6]. ERP recordings were performed at least 90 days after injury. All patients had a documented presence of the auditory startle (i.e., CRS-R auditory subscale score ≥ 1) or the auditory N1 ERP component, indicating intact hearing. None had a documented history of prior brain injury or premorbid hearing impairments, and no sedation was given within 24 hours prior to the recording.

Two controls and four patients were excluded due to low quality EEG recordings (i.e., ocular, muscle, and/or noise artifacts that could not be adequately corrected for by the preprocessing procedures). Hence, 20 controls (mean age = 38, range 25–61 years; 10 males) and 20 patients (mean age = 40, range 19–66 years; 11 males) were included in the ERP analysis. The control group was comparable to the patients with regard to gender and age distribution. Nine patients were classified as MCS+ and eleven were MCS− according to the CRS-R scores obtained by an experienced rater on the day of EEG recordings (Table 1). MCS+ was defined as presence of reproducible response to command (CRS-R auditory subscale score ≥ 3) and MCS− as no reproducible response to command (CRS-R auditory subscale score ≤ 3 [40]). Diagnostic distinction between MCS+ and MCS− was hence derived from the complexity of present behavior on the auditory subscale.

The study was conducted in agreement with the Helsinki Declaration and was approved by the Regional Committee for Medical Research Ethics in South East Norway. Written informed consent was obtained from healthy controls and the patients' next of kin.

### 2.2. Experimental Procedures

**Figure 1:**
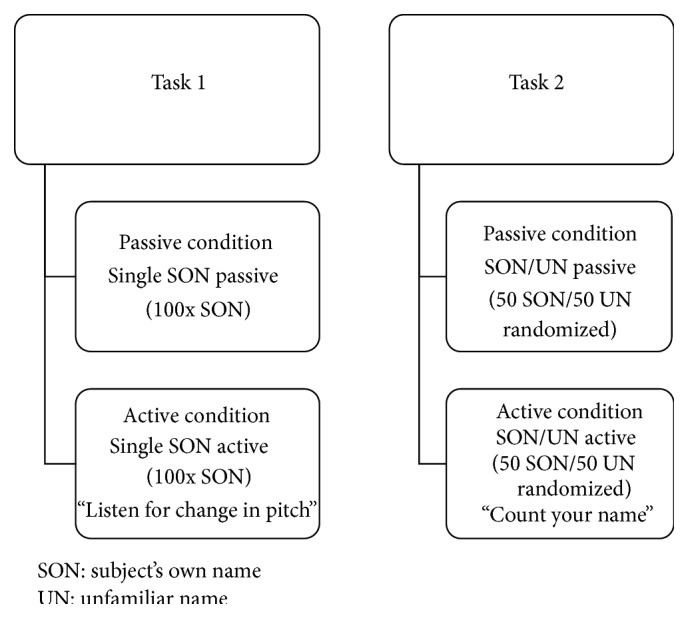
Experimental ERP design.

The ERP paradigm consisted of two tasks, both containing a passive and an active condition (Figure 1). All four conditions were presented in the same hierarchical order (each condition containing four sets of consecutive blocks of 25 stimuli, 100 in total). Task 1 consisted of single SON passive and active conditions, identical to the design used in Schnakers et al.'s latest study [30]. The single SON passive condition contained SON repeated 100 times, with the instruction to do nothing but to stay awake. Thereafter, the subjects were presented with the single SON active condition, with the instruction to listen very carefully for a change in the pitch of the voice saying their name. There was no actual change in the voice, rendering the physical stimulus characteristics identical, and the demanded level of mental effort was the only difference between conditions. Task 2 included two-stimuli SON/UN passive and active conditions, where SON (*n* = 50) was randomly interspersed in between an unfamiliar name (UN—confirmed unfamiliar by family or the healthy controls themselves, *n* = 50). In the passive condition, the subjects were instructed to do nothing but to stay awake. In the active condition, subjects were instructed to count the number of times they heard SON, requiring sustained attention and working memory effort. Hence, the active conditions in tasks 1 and 2 differed with regard to cognitive load. Instructions were repeated between each block of 25 stimuli for all conditions. EEG recordings were performed while participants were in a wakeful state. For the patients, a short break and, if needed, brief auditory or deep pressure stimulation according to CRS-R protocol were applied between conditions in order to ensure adequate arousal levels. All names were digitally recorded from a female, middle-aged native Norwegian speaker (stimulus duration range: approximately 500–600 ms), and a stimulus onset asynchrony of 2000 ms was used.

### 2.3. EEG Acquisition

Data were acquired at the patients' bedside with a 32-electrode cap (Quik-Cap; Compumedics Neuroscan) connected to a portable digital NuAmp EEG amplifier (Compumedics Neuroscan). Electrooculogram was recorded using the electrodes located above and below the left eye and at the outer canthi of the two eyes. The ground electrode was placed near Fz and a nasal reference was applied. The EEG signals were acquired using the NeuroScan Inc. acquisition unit with an analog band-pass filter of 0.1 to 200 Hz and a sampling frequency of 500 Hz. The impedance was kept below 10 kΩ. Auditory stimuli were presented binaurally through earphones with a maximum 90 dB sound pressure level. The whole procedure lasted approximately 25–30 minutes including breaks.

### 2.4. Behavioral Assessment of DoC Patients

The Coma Recovery Scale-Revised (CRS-R) is comprised of six subscales addressing auditory, visual, motor, oromotor/verbal, communication, and arousal functions [41]. Items are hierarchically arranged, from reflexive to cognitively mediated responses. The lowest item score on each subscale represents reflexive activity, whereas the highest scores reflect cognitively mediated behaviors. The scale has good psychometric properties and is sensitive for behavioral assessment of DoC [13, 41, 42]. The authorized Norwegian version was used on the day of the EEG recording. A validation study of the authorized Norwegian version has confirmed acceptable psychometric properties comparable to the original CRS-R [43].

### 2.5. ERP Analysis

EEG data were analyzed with custom-made MATLAB (The MathWorks, Inc., Natick, MA, USA) scripts built on the open source EEGLAB environment (http://sccn.ucsd.edu/eeglab/) [44] and the study function in EEGLAB. Data were high-pass-filtered above 1 Hz. Artifact correction was performed on epoched data (−500 to 1500 ms) by excluding independent components (ICs) characteristic of nonbrain artifact (e.g., eye, muscle, or line noise) identified by inspection of topographies, time courses, and activity spectra. Following artifact removal, data were low-pass-filtered below 20 Hz. Bad channels were interpolated and trials with amplitude values exceeding ±75 *μ*V were rejected at the midline electrodes Fz, Cz, and Pz. Average activity between −200 and 0 ms was defined as baseline.

In order to investigate the robustness of a design comparing stimulus type and two different active conditions in healthy controls, grand-averaged ERPs were computed for SON and UN as well as for the active and passive condition in both tasks. Peak and mean amplitudes over specified time windows were exported for statistical analyses. Peak amplitudes for the N1 component were derived from the 80–150 ms time window at Cz, corresponding to the expected latency and distribution for the auditory N1 wave [45, 46]. Based on grand average group ERPs, a temporal window between 500 and 680 ms after stimulus was determined for extracting peak and mean P3 amplitudes for SON in the active and the passive conditions in task 1 and correspondingly between 300 and 500 ms for SON and UN in task 2.

The second aim was to investigate P3 responses in the MCS+ and MCS− patient groups with regard to active mental tasks on an individual level and also to explore the robustness of the tasks on an individual level in the control group. The P3 component was considered present on an individual level when detected in the expected time window at all three midline electrodes (Fz, Cz, and Pz), by means of visual inspection by three of the authors (Solveig L. Hauger, Marianne Løvstad, and Stein Andersson). Subjects with consensus-based identification of larger P3 amplitude values in the active versus passive condition at minimally one electrode were defined as “responders.” One of the authors was blinded to the CRS-R results, while the other two were involved in both CRS-R scoring and ERP analyses.

### 2.6. Statistical Analyses

In the healthy control group, averaged ERP amplitudes were subject to statistical analysis, using SPSS version 22 for Macintosh (SPSS, Inc., Chicago, IL). Repeated measures analysis of variance (ANOVAs) was used to examine differences in mean or peak amplitude between stimulus types or conditions in the healthy control group. Either SON versus UN or active versus passive condition was contrasted, with stimulus type (SON-UN) or condition (passive-active) as within-subjects factors and electrode location along the midline (Fz, Cz, and Pz) as the second within-subject factor. Extreme values were identified using boxplots. Analyses including extreme values were repeated without these, and any resulting changes in results are reported. Greenhouse-Geisser epsilon corrected *p* values are reported for computations involving more than two levels of a repeated measures factor. When indicated by the ANOVA, post hoc tests with Bonferroni correction were run. Partial eta squared (partial *η* ^2^) was used to calculate the sample effect size based on within-subjects factor variability. Effect size values of .01, .06, and .14 have been suggested to represent small, medium, and large effect sizes, respectively, but larger values will often be expected in nonsocial experimental research, such as physiology [47–49].

To investigate if each visually identified responder could be confirmed statistically, an unpaired *t*-test was performed in the EEGLAB study function [44]. Amplitude differences between passive and active conditions were tested on an individual level on a trial-by-trial basis for each sampling point, with Bonferroni correction for multiple comparisons. Rate of responders is described by actual numbers of subjects, percentage, and 95% confidence interval (CI). Statistical significance was set to *p* < .05. Despite the small sample and thus a need for caution in interpreting results, sensitivity and specificity of the ERP tasks were calculated with CRS-R as the reference standard and MCS− as the disorder of interest.

## 3. Results

### 3.1. The Effect of SON versus UN and Active versus Passive Experimental Conditions in Healthy Controls

**Figure 2:**
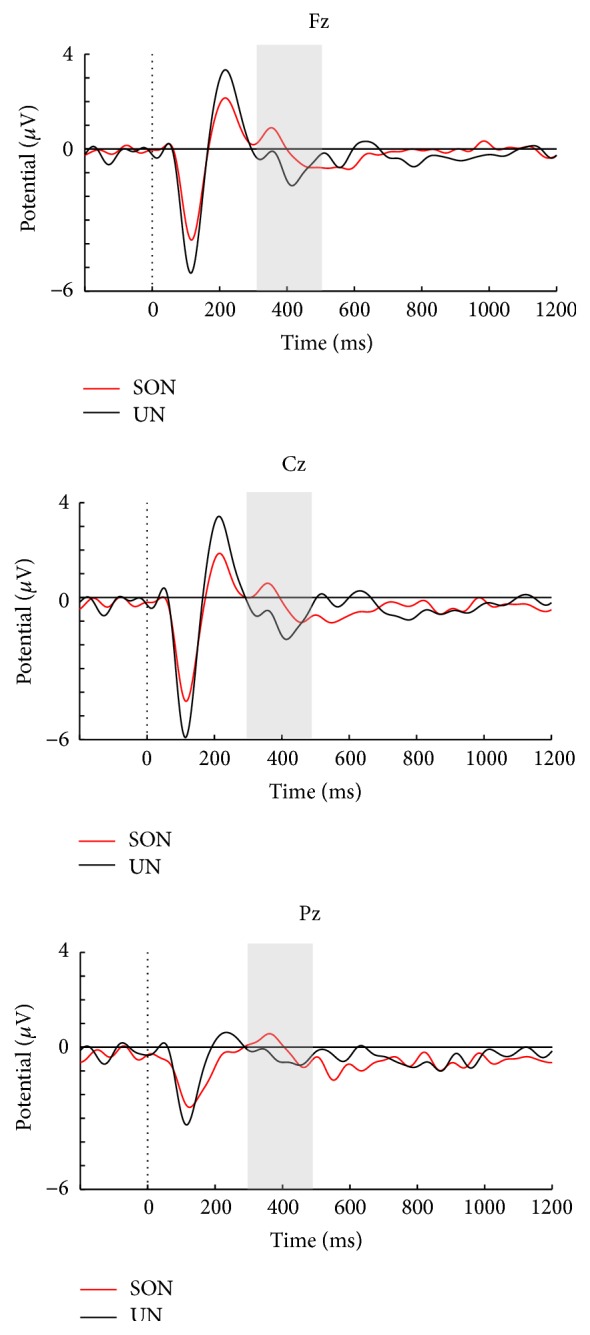
Stimuli SON and UN in passive listening task for the healthy controls.

**Figure 3:**
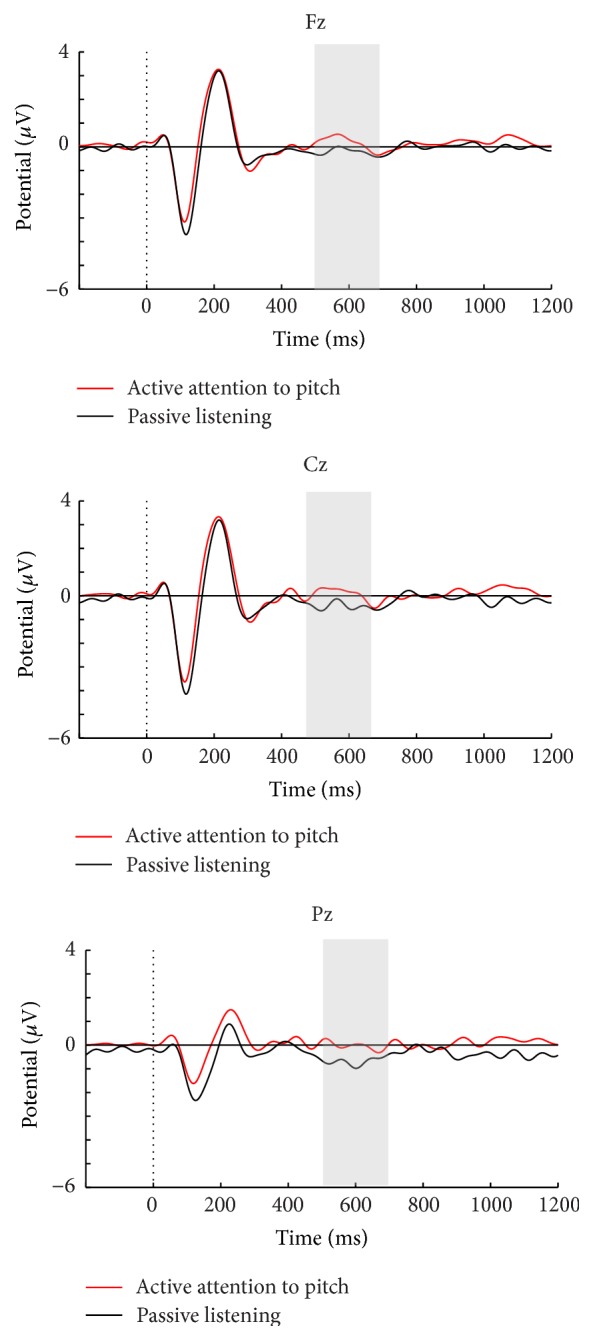
Passive and active conditions in task 1 for the healthy controls.

**Figure 4:**
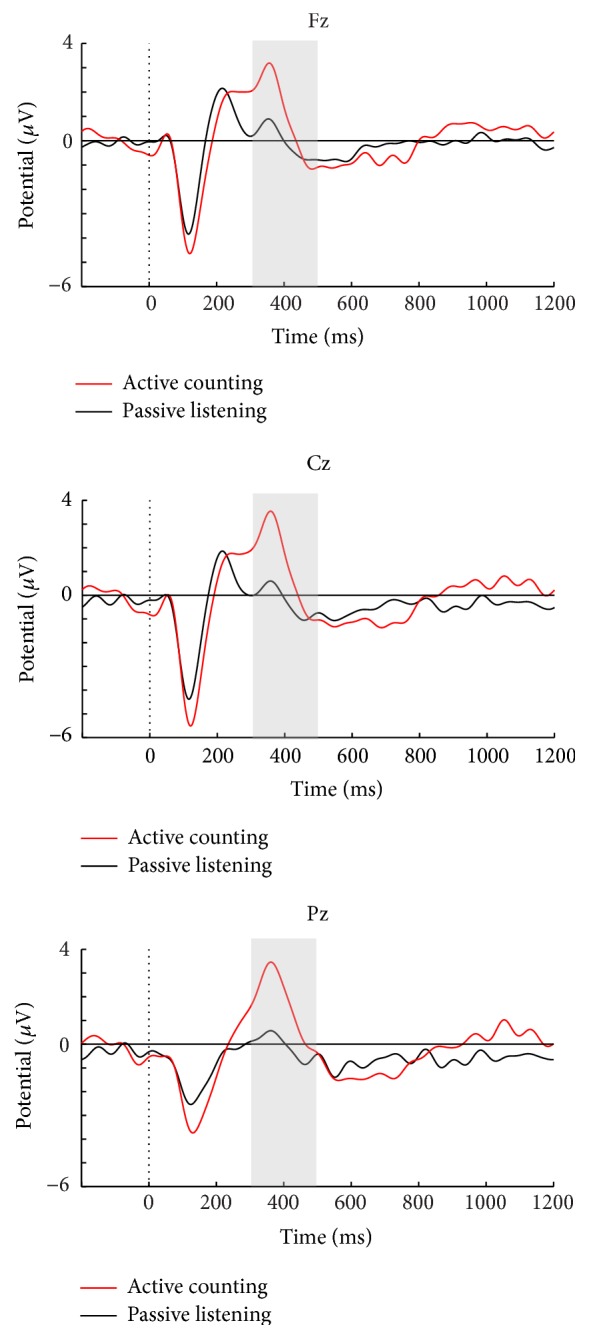
Passive and active conditions in task 2 for the healthy controls.

Grand average ERPs for SON versus UN in a passive listening task as well as passive versus active conditions in tasks 1 and 2 are presented in Figures 2, 3, and 4. Visual inspection suggested that SON generally elicits a larger P3 compared to UN (Figure 2). The active conditions in both tasks elicited a larger P3 for SON compared to the passive conditions, but with larger amplitude differences in the counting condition of task 2 (Figures 3 and 4). Moreover, visual inspection suggested that the counting condition in task 2 elicited larger N1 amplitudes compared to task 1.

#### 3.1.1. Effects of SON and UN on N1 and P3

A main effect of stimulus type in task 2 reflected that the control group had a larger N1 peak amplitude at Cz for UN compared to SON *F*(1, 19 = 6.42, *p* = .02, and partial *η* ^2^ = .25).

We also found that the type of name presented had significant effects on P3 amplitude in the control group, with larger amplitudes to SON compared to UN in the passive condition of task 2 for both peak (*F*(1, 19 = 4.45, *p* = .048, and partial *η* ^2^ = .19)) and mean amplitudes (*F*(1, 19 = 5.56, *p* = .03, and partial *η* ^2^ = .23)). A significant effect of electrode location was found for the mean amplitude analysis (*F*(1.45, 27.58) = 8.51, *p* = .003, and partial *η* ^2^ = .31), due to a larger P3 at Pz compared to Fz (*p* < .001).

#### 3.1.2. Effects of Active Task Instructions on N1

The auditory N1 component elicited by the passive and active conditions in task 1 did not differ in peak amplitude at Cz. However, as Figure 4 shows, enhancement of N1 was detected at Cz in the active counting compared to passive listening in task 2 (*F*(1, 19) = 6.85, *p* = .02, and partial *η* ^2^ = .27). Moreover, a significant difference was found when comparing the active conditions in tasks 1 and 2, with a larger N1 in the counting task (*F*(1, 19) = 15.68, *p* < .001, and partial *η* ^2^ = .45).

#### 3.1.3. Effects of Active Task Instructions on P3

The active condition in task 1 elicited a significantly larger mean P3 amplitude (*F*(1, 19) = 6.03, *p* = .02, and partial *η* ^2^ = .24), compared to the passive condition (see Figure 3). This was however not significant in the peak analysis. A significant main effect of electrode location was evident for mean amplitudes (*F*(1.35, 25.67) = 10.52, *p* = .002, and partial *η* ^2^ = .36), due to a larger amplitude at Fz compared to both Cz (*p* = .04) and Pz (*p* = .01).

In task 2, the instruction to count SON resulted in a significant main effect of condition, with a larger P3 compared to passive listening to SON for both peak (*F*(1, 19) = 44.83, *p* < .001, and partial *η* ^2^ = .78) and mean amplitudes (*F*(1, 19) = 24.44, *p* < .001, and partial *η* ^2^ = .56). No significant main effect of electrode location in peak was found, but there was for mean amplitude (*F*(1.22, 23.12) = 5.32, *p* = .03, and partial *η* ^2^ = .22), due to a maximum effect at Pz compared to both Fz (*p* = .03) and Cz (*p* = .02). Likewise, P3 to SON in the counting condition was also significantly larger than that to SON in the passive condition in task 1 (*F*(1, 19) = 32.12, *p* < .001, and partial *η* ^2^ = .63), with a main effect of electrode location (*F*(1.16, 22.01) = 7.15, *p* = .01, and partial *η* ^2^ = .27), due to a maximum effect at Pz compared to Fz (*p* = .03).

### 3.2. Individual Responders among Controls and Patients in Active Tasks

#### 3.2.1. Individual P3 Effects in Controls

A main objective was to identify the rate of individual responders, that is, subjects with elevated P3 amplitudes in active compared to passive conditions. As noted in Table 2, 15/20 controls showed an enhanced P3 in the active compared to the passive condition of task 1. On a trial-by-trial basis, 11 of these could be confirmed statistically. On the other hand, all controls, except one, displayed larger P3 amplitudes in the active counting condition of task 2 (see Table 2). Here, enhanced P3 curves in the counting relative to the passive listening condition was confirmed statistically on an individual level in 17/20 controls.

#### 3.2.2. Individual P3 Effects in MCS Patients

**Figure 5:**
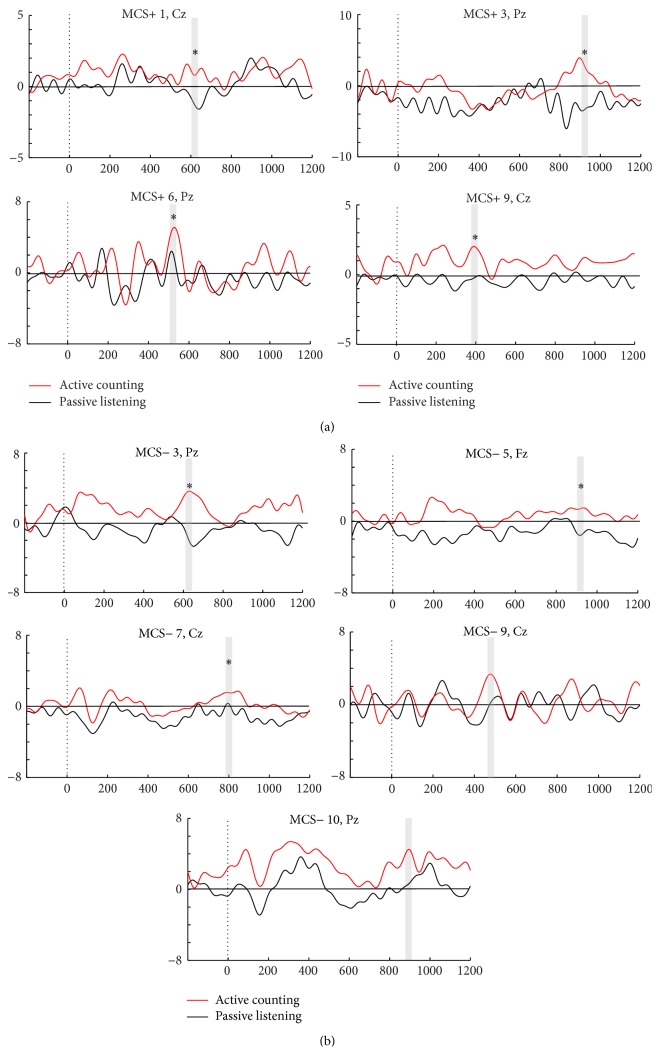
Individual patients considered responders in the active counting condition of task 2. (a) Illustrates ERPs in individual MCS+ responders in task 2, while (b) provides ERPs in individual MCS− responders. The averaged ERPs in the active counting (red) versus passive (black) condition (*y*-axis, amplitude in *μ*V; *x*-axis, time in ms) are illustrated. Observed significant differences of P3 amplitude between conditions (*p* values < .05 to .001) are marked with a star above the P3 curve marked with grey line.

The criterion for a patient being identified as a responder was the same as for the healthy controls, namely, identification of the P3 component at all midline electrodes and a larger P3 at minimally one midline electrode in the active condition compared to the passive. On visual inspection, only four patients (three MCS+/one MCS−) showed a larger P3 component in the active compared to the passive condition of task 1 (see Figure 1), whereas three (all MCS+) could be confirmed statistically. Also, six MCS+ patients who demonstrated command-following behaviorally failed to be detected in task 1, rendering a false negativity rate of 67%, while one MCS− patient with absence of behavioral command-following was considered a responder in this task. On the other hand, 9/20 patients (four MCS+/five MCS−) showed higher P3 amplitudes in the active counting condition compared to the passive listening in task 2. Here, five MCS+ patients who showed behavioral command-following did not display elevated P3 responses in the counting condition, yielding a false negative rate of 56%. Yet, five MCS− patients with absence of behavioral command-following were considered responders in this task. Seven of these identified responders (four MCS+/three MCS−) could be confirmed statistically in a trial-by-trial basis analysis. The MCS responders in the counting task are shown in Figure 5, where ERPs are illustrated at the midline electrode with the most pronounced P3 response. Notably, only two patients (patient MCS+ 1 and MCS− 10) were responders across both active tasks, and therefore a total of 11/20 MCS patients had enhanced P3 amplitude in one of the two active counting conditions, or both. All in all, 5 patients showed elevated P3 in active mental tasks (in one or both tasks), but no behavioral command-following. Sensitivity of the ERP assessment was 67% (95% CI ±30.7), and as six MCS− patients also lacked a P3 effect in the active tasks, specificity was 55% (95% CI ±29.4). In addition, of the nine patients that showed command-following in the CRS-R assessment session, three failed to demonstrate enhancement of P3 in either active condition.

#### 3.2.3. P3 Effects in the Active Counting versus Passive Condition of Task 2 in MCS Responders

The patient responders' grand average ERPs in task 2 were investigated with the same procedure as the healthy control group data (temporal window for analysis of P3 mean amplitude in patient responders was examined at both 300–500 and 800–1000 ms after stimulus). As can be seen in Figure 5, there was a prominent heterogeneity in P3 latency across the individual patient responders, rendering a lack P3 effect of the active counting condition on a group level.

There was no significant difference in time since injury between patients classified as responders in task 1 or 2 and nonresponders.

## 4. Discussion

The aim of this study was to explore the robustness of two active ERP tasks that differed in stimulus type and cognitive load. Tasks were first administered to healthy subjects and then to a group of MCS patients to investigate their utility in detecting covert command-following on an individual level.

To probe for covert cognitive resources in DoC patients with electrophysiological methods requires specially tailored stimuli with an established probability of eliciting P3 responses. This study used the subjects' own name (SON) contrasted with an unfamiliar name (UN) and revealed a larger P3 to SON compared to UN in the control group, confirming earlier findings showing increased probability of enhanced responses with salient self-referential stimuli [25–27, 29, 38]. A larger N1 was in contrast found to UN. N1 is recognized to be affected by stimuli change and attention [45, 50]. At the time point control subjects are introduced to UN, they have already been exposed to SON repeatedly. Herein, the addition of UN to SON represents a novelty of UN, and attention allocation towards novel stimuli may thus have affected the early N1 component in controls.

In the inquiry of which of the two ERP tasks constitutes the most robust paradigm among healthy controls, both a markedly larger N1 and P3 potential were found for the active task requesting counting of SON compared to active listening to pitch in the control group. The robustness of the counting task was furthermore confirmed when exploring the rate of individual responders across the two tasks among the healthy subjects, revealing a 95% responder rate in the counting task. In contrast, only 75% of the healthy controls could be identified as responders in the task where listening for a change in pitch constituted the active task. This is in line with the previous study of Schnakers and colleagues using the same pitch paradigm, where a 78% responder rate was seen in the control group [30]. Taken together, the results suggest greater robustness of the counting task in eliciting a P3 effect in active tasks. The robustness of tasks requiring counting of a target stimulus has also been demonstrated in earlier DoC-related ERP studies, showing a 100% responder rate amongst individual healthy controls [37, 38]. The robustness of the counting task was also reflected in the patient group, with more responders in the counting task (9/20) compared to actively listening for change in pitch (4/20). Although it is established that P3 amplitude is affected by level of attentional task load [22, 23], it is not the actual P3 amplitude, but the fact that a difference between active and passive tasks can be identified indicates the presence of consciousness on an individual patient level. Instruction to actively listen for change in pitch in task 1 elicited a more pronounced frontal P3 effect in the control group. This result is comparable with the frontal P3 effect found in the previous study of Schnakers et al. [30], using the same pitch paradigm. In contrast, the counting task elicited a larger parietal P3 effect. The differentiation in P3 topography elicited in the control group between the two active task instructions most likely reflects divergence in the attentional demands of the task. Actively listening for change in pitch represents a low cognitive load but requires focused attention, while the counting task demands working memory and selective attention towards SON. The results are in line with previous ERP studies that have also found a parietal P3 effect in healthy controls and MCS patients when instructed to count a specific target stimulus [39, 51], and a parietal activation is furthermore described as the typical scalp distribution of a target P3 response with working memory load [22].

With regard to the ability of ERP to distinguish between MCS+ and MCS−, sensitivity was 67%, with three patients with definite behavioral signs of consciousness not displaying clear electrophysiological evidence of command-following. Previous studies have also revealed false negatives, although these numbers are not always reported. The lack of responses in ERP tasks could be explained by a number of cognitive factors, such as variability in vigilance and arousal, fatigue, habituation, and limited attention span. However, lack of enhanced P3 response in active tasks in the current study cannot solely be explained by fatigue, habituation, or decreased arousal levels over time, as there were more responders in the last condition. However, fluctuations in vigilance throughout the session cannot be ruled out. Additionally, of the MCS+ patients, only 6/9 showed command-following both in ERP and CRS-R, demonstrating inconsistency between the behavioral and neurophysiological measures. This could either be explained by fluctuation of functioning or the fact that one could argue that the ERP experiments of active listening to pitch or counting represent a higher cognitive demand compared to the command-following instructions of the CRS-R, for example, to move a limb, or look at a target stimuli. The ERP tasks require the patient not only to be awake and conscious during the recording, but also to understand the instructions, be able to keep perceptual representations in working memory, and continuously perform the task. In other words, a DoC patient with cognitive impairments in any of the listed processes may miss the task but do not lack consciousness. Furthermore, the response is assessed over a shorter number of sequences in the CRS-R compared to ERP. In summary, also MCS+ patients are likely to suffer from underlying severe cognitive deficits, and the probability of revealing their residual cognitive resources in ERP assessment is dependent upon the complexity of the tasks. Hence, as previous fMRI studies have emphasized, negative findings in this patient group cannot be interpreted as evidence that the patients lack awareness or cognitive abilities [19, 52, 53]. A goal is to establish ERP tasks that are demanding enough to elicit cognitively mediated responses, but simple enough to not exceed the cognitive capacity of severely brain damaged individuals.

On the other hand, a specificity of only 54% was due to 5 MCS− patients displaying electrophysiological, but not behavioral signs of command-following. As there is no established veridical benchmark of level of consciousness [8, 54–56], the relatively low specificity numbers can be explained by the small sample or might actually be due to the fact that behavioral measures such as the CRS-R in some cases do not detect the true level of functioning in the patient. The results are in line with previous ERP studies using SON to detect covert cognitive resources in DoC patients. In Schnakers and colleagues' recent study [30], 3/8 MCS− and 1/10 VS displayed electrophysiological signs of higher cognitive functioning, undetected by standardized behavioral assessment. Also, in their earlier study, covert signs of command-following were detected in 2/6 MCS patients [38]. Signs of covert residual cognition were also detected in Lulé et al.'s study, but instead of using a salient stimulus, they instructed participants to count a target “yes” or “no” [36]. Of the 13 MCS patients included, only one, also lacking behavioral signs of command-following, could do the ERP task, but none of the three VS patients. Taken together, these studies suggest that functional neurophysiological methods may aid in detecting volitional cognition in a minority of patients where this is not accomplished with behavioral scales such as the CRS-R. Thus, this study provides further evidence that the absence of behavioral signs of cognition in severely brain-injured patients does not always indicate the true absence of such abilities and that counting the salient stimuli SON as an active ERP task may facilitate detection of covert residual cognition in DoC.

The P3 component is thought to be produced by either multiple, relatively independent generators or reflection of a central integrated system with widespread connections and impact throughout the brain. Preserved parts of this complex cortical and subcortical system may thus still enable the capacity to generate P3 [20]. However, there is debate as to whether the recordings of P3 amplitude could be affected according to different etiologies of brain injury. Cruse et al. [57] investigated the difference between TBI and non-TBI etiology regarding both overt and covert cognitive capacity in a group of MCS patients. In their EEG study, 3/4 TBI MCS patients who could not follow commands behaviorally demonstrated evidence of cognitive processing on an active imagery task, compared with none of the four non-TBI MCS patients. The authors argued that patients who progress to the MCS after a non-TBI are significantly less likely to produce evidence of high-level cognitive functioning than traumatically injured MCS patients. We did not observe a difference in detectable covert cognition based on injury mechanism in the present study. Among the 11 MCS patients showing enhanced P3 amplitude in one or both of the active counting conditions, five had TBI and six non-TBI. Of the five patients showing elevated P3 in active mental tasks, but no behavioral command-following, two were non-TBI. Additionally, no link was found between responders and nonresponders with regard to time since injury.

In order to incorporate new functional neurophysiological techniques in addition to standardized behavioral assessment in the clinical setting, establishment of robust electrophysiological measures of brain activity is required. In accordance with the results of this study, the robustness of counting SON contrasted to a passive condition increases the detection of residual covert cognitive resources on an individual level in patients with disordered consciousness and thus may provide valuable complementary clinical information in a subset of patients. A P3 response in healthy persons typically peaks between 300 and 600 ms [22]; however, prolonged latency in brain injured patients relative to healthy subjects has been demonstrated [58], also when using the persons' own name [26, 30, 38]. The results show that P3 latency varies prominently between individual patients, resulting in an inconsistent P3 effect for the active counting condition on the MCS group level. Thus, in order for this ERP paradigm to be a robust and sensitive measure of covert command-following in MCS patient groups, analysis procedures must adapt to individual, and often prolonged, P3 latencies.

*Study Limitations*. This study illustrates several challenges concerning ERP studies in patients with DoC. Inherent to the DoC diagnosis, nonsedated DoC patients cannot reliably follow simple instructions. This includes difficulty following instructions not to move the eyes and body during EEG recordings, often leading to excessive motor artifacts that need to be addressed carefully in the preprocessing analysis. Thus, one would expect the signal-to-noise ratio to be lower than in healthy controls, further confirming the need for robust experimental paradigms. Furthermore, the high false negative rate we observed may be due, in part, to the fact that only one ERP recording was performed per patient. The importance of conducting serial reassessments with standardized behavioral scales [9, 59] also applies to other methodologies, including ERP. Thus, multiple ERP assessments performed on the same day or on different days would potentially reduce false negative ERP findings, but this is time-consuming and may not be realistic in a clinical setting. In future research there is a need to investigate retest reliability of ERP responses to counting SON. Also, while the ERP method has a great advantage in bypassing requirements for coordinated motor output, DoC patients may have underlying cognitive difficulties in understanding instructions limiting their capacity to engage in active ERP tasks. It has been suggested that when assessing DoC patients, one should take into account potential language deficits and provide adaptive accommodations such as presenting written or gestural instructions [60]. Further effort is required in developing robust test procedures that are not restricted by language comprehension. While neurophysiologic studies are subject to many of the same constraints as neuroimaging and behavioral studies [55], ERP may detect conscious awareness in patients who would otherwise be missed by alternate assessment modalities. Finally, the restricted sample size calls for interpretive caution regarding the exact specificity and sensitivity estimates.

## 5. Conclusion

To date, neurophysiological studies of residual cognitive capacity in DoC patients have been conducted with limited knowledge about which type of cognitive tasks constitutes the most robust paradigm when it comes to eliciting electrophysiological indices of covert cognitive capacity on an individual patient level. This study confirms that the use of an active task of counting the subjects' own name contrasted to a passive listening task is robust in probing for volitional cognitive capacity in MCS patients. In spite of the fact that clinical ERP assessment on an individual level in DoC patients is challenging, it offers supplementary information about covert cognitive resources in some patients.

## Figures and Tables

**Table 1:** Patients' diagnosis, etiology, gender, age, time since injury, and CRS-R scores.

Patients	Etiology	Sex	Age	TSI months	CRS-R total	AF	VF	MF	OF	C	Ar
MCS− 1	TBI	M	34	115,9	10	1	3	2	2	0	2
MCS− 2	TBI	M	34	57,7	15	2	4	5	2	0	2
MCS− 3	TBI	M	19	63,6	7	1	3	1	1	0	1
MCS− 4	TBI	F	66	6,0	10	2	3	2	2	0	1
MCS− 5	TBI	F	19	5,3	13	1	3	5	2	0	2
MCS− 6	Anoxia	F	29	6,5	10	2	3	2	1	0	2
MCS− 7	TBI	M	27	40,2	11	2	3	2	2	0	2
MCS− 8	TBI	M	29	39,0	8	0	0	4	2	0	2
MCS− 9	Anoxia	M	54	9,5	8	1	3	2	1	0	1
MCS− 10	Encephalitis	M	49	4,3	11	2	3	2	2	0	2
MCS− 11	TBI	M	47	4,8	13	2	3	5	2	0	1
MCS+ 1	SAH	F	49	56,1	11	3	3	2	2	0	1
MCS+ 2	TBI	F	24	47,0	14	3	3	2	2	1	2
MCS+ 3	TBI	F	35	117,0	12	3	3	2	2	0	2
MCS+ 4	TBI	M	60	29,1	18	4	4	5	2	1	2
MCS+ 5	Anoxia	M	50	3,6	15	3	3	5	2	0	2
MCS+ 6	TBI	M	35	4,3	18	3	5	5	2	1	2
MCS+ 7	Encephalitis	F	27	6,8	9	3	1	2	1	0	2
MCS+ 8	TBI	F	58	8,8	13	3	5	2	1	1	1
MCS+ 9	SAH	F	49	29,0	8	3	0	2	1	0	2

MCS−: minimally conscious state minus; MCS+: minimally conscious state plus; TBI: traumatic brain injury; SAH: subarachnoidal hemorrhage; M/F: male/female; TSI: time since injury (months after injury); CRS-R: Coma Recovery Scale-Revised; AF: auditory function; VF: visual function; MF: motor function; OF: oromotor function; C: communication; and Ar: arousal.

**Table 2:** Controls and patients classified as responders in tasks 1 and 2.

Group	Task 1				Task 2			
	Yes^*∗*^		No^*∗∗*^		Yes		No	
CTR (*N* = 20)	15	75% (CI = ±19.0)	5	25%	19	95% (CI = ±9.6)	1	5%
MCS (*N* = 20)	4	20% (CI = ±17.5)	16	80%	9	45% (CI = ±21.8)	11	55%
MCS+ (*N* = 9)	3	33% (CI = ±30.7)	6	67%	4	44% (CI = 32.4)	5	56%
MCS− (*N* = 11)	1	9% (CI = ±16.9)	10	91%	5	45% (CI = 29.4)	6	55%

^*∗*^Yes = subjects identified as responders in active condition.

^*∗∗*^No = subjects identified as nonresponders in active condition.

CI = 95% confidence interval.
